# New Appliances and Things Medical

**Published:** 1905-02-04

**Authors:** 


### NEW APPLIANCES AND THINGS MEDICAL.

### CREAM EMULSION OF COD-LIVER OIL, COMBINED WITH HYPOPHOSPHITES OF LIME AND SODA.

(Adley, Tolkein and Co., Limited, Novas Chemical Works, Blackburn.)

This preparation is fairly free from taste and odour and has all the characteristics of an oil prepared either during the life or immediately after the death of the fish, and seems to be free from oxidation products. We are glad to see a comparatively new preparation of cod-liver oil on the market, for recently there has been some difficulty in obtaining an adequate supply. Although cod-liver oil should consist to the extent of 98·9 per cent. of olein, its other constituents, especially the phosphorus, the iodine, chlorine, and bromine, exert an action which quite distinguishes it from other fats. Experience has shown that hypophosphites can be well combined, as in the present instance, with cod-liver oil.

### PERRIER WATER.

(45 and 46 New Bond Street, W.).

This water is a natural mineral water, occurring at Vergèze, in France. The water contains a relatively high proportion of $CO_2$, but, so far as we have heard, it does not give rise to any gastric distention or other inconvenience often attending the taking of highly effervescing water. This appears to depend upon the fact that all the gas in the water occurs naturally, and the water is not saturated artificially with gas. The taste of the water is pleasant and sharp, and it goes well with all wines and spirits, and also with milk. In this connection we would emphasise the fact that a very small quantity of it, when added to milk that has been boiled, immediately destroys the somewhat flat and insipid flavour of boiled or Pasteurised milk. It is alkaline in reaction, contains ·0941 solid matter to the litre, which consists of potash, calcium, sodium, magnesium, and aluminium salts.

### DROITWICH BRINE CRYSTALS.

(Weston and Westall, Limited, 41 Eastcheap, London, E.C.).

The value of Droitwich brine baths in certain forms of rheumatism is undoubted, and these baths, in spite of the introduction of many new and efficacious anti-rheumatic remedies, have held their position. The attractions, however, of Droitwich as a place, in spite of many improvements, are not great, and the possibility of having the Droitwich baths at home will appeal to many doctors and patients. The brine crystals are intended for this purpose, and to make an average Droitwich bath, 2 lb. of them should be added to each gallon of water.

